# Case Report: Post-mortem analysis of long-term inflammation after mild COVID-19

**DOI:** 10.3389/fimmu.2026.1913045

**Published:** 2026-08-27

**Authors:** Yang Wu, Yu Kit Chan, Denise Goh, Belinda Lee, Felicia Wee, Mai Chan Lau, Paul Chui, Thuan Tong Tan, Jabed Iqbal, Akhila Balachander, Craig Ryan Joseph, Lisa F.P. Ng, Yee Sin Leo, Joe Yeong

**Affiliations:** 1 Institute of Molecular and Cell Biology (IMCB), Agency for Science, Technology and Research (A*STAR), Singapore, Singapore; 2 National Centre for Infectious Diseases, Singapore, Singapore; 3 Department of Infectious Diseases, Tan Tock Seng Hospital, Singapore, Singapore; 4 Forensic Medicine Division, Health Sciences Authority, Singapore, Singapore; 5 Bioinformatics Institute (BII), Agency for Science, Technology and Research (A*STAR), Singapore, Singapore; 6 Department of Infectious Diseases, Singapore General Hospital, Singapore, Singapore; 7 Department of Anatomical Pathology, Singapore General Hospital, Singapore, Singapore; 8 Singapore Immunology Network (SIgN), Agency for Science, Technology and Research (ASTAR), Singapore, Singapore; 9 Integrated Central Operations and Research Enablers (ICORE), Agency for Science, Technology and Research (A*STAR), Singapore, Singapore; 10 Infectious Diseases Labs (ID Labs), Agency for Science, Technology and Research (A*STAR), Singapore, Singapore; 11 Lee Kong Chian School of Medicine, Nanyang Technological University, Singapore, Singapore; 12 Saw Swee Hock School of Public Health, National University of Singapore, Singapore, Singapore; 13 The First Affiliated Hospital of Wenzhou Medical University, Wenzhou, China; 14 School of Basic Medical Science, Wenzhou Medical University, Wenzhou, China; 15 Division of Cancer Signalling and Therapy, Institute of Molecular and Cell Biology (IMCB), Agency for Science, Technology and Research (A*STAR), Singapore, Singapore; 16 Division of Pathology, Singapore General Hospital, Singapore, Singapore; 17 Zhejiang Key Laboratory of Intelligent Cancer Biomarker Discovery and Translation, The First Affiliated Hospital of Wenzhou Medical University, Wenzhou, China; 18 Duke-NUS Medical School, Singapore, Singapore; 19 Department of Microbiology, Singapore General Hospital, Singapore, Singapore

**Keywords:** immune dysregulation, macrophage, post-acute COVID-19 syndrome (PACS), post-mortem cohort, SARS-CoV-2, spatial transcriptomics, tissue inflammation, viral persistence

## Abstract

Persistent inflammation and tissue injury may contribute to post-acute COVID-19 syndrome (PACS). However, direct tissue evidence from mildly-infected individuals sampled long after recovery remains exceptionally limited. Here, we performed post-mortem bulk RNA-seq and GeoMx digital spatial profiling of lung, heart, and brain tissues from four individuals who recovered from mild COVID-19 more than 250 days before death from unrelated causes. Samples were collected during the early phase of the national vaccination program, before widespread vaccine uptake and recurrent infection, providing a rare reference cohort for defining tissue responses to SARS-CoV-2 in the absence of major confounding from vaccination and/or reinfection; such a cohort can no longer be prospectively assembled. We observed residual SARS-CoV-2 nucleocapsid antigen within tissues and revealed organ-specific immune alterations. Lung and heart tissues exhibited a coordinated, self-sustaining inflammatory response, whereas brain tissue showed vascular dysfunction and altered neuroimmune homeostasis. These findings showcase the distinct tissue responses following mild infection and provide a spatially resolved view of sub-clinical long-term tissue sequelae. Collectively, our data establish a unique baseline for persistent viral antigen and tissue-specific immune dysregulation underlying PACS.

## Introduction

Post-acute COVID-19 syndrome (PACS) is characterized by prolonged symptoms and multisystem effects after acute SARS-CoV-2 infection. Proposed mechanisms include continued immune cell activation with elevated cytokines and viral persistence in tissues (1, 2). Recent tissue-level evidence strengthened the case for prolonged viral residence. SARS-CoV-2 RNA was detected in gut tissue up to 676 days after infection (3), and viral persistence was demonstrated in 10 organs, which was associated with a 5-fold increased odds of long-COVID symptoms in a cross-sectional Chinese cohort (4). These studies evaluated patients in a contemporary, vaccinated, reinfection-rich settings and/or those who were symptomatic at the time of biopsy. Although highly relevant to current clinical practice, analysis of pre-vaccination cohort provides a unique reference for characterizing defining unmodified disease pathogenesis, free from confounding effects of vaccination and repeated infection. However, due to the near-universal vaccination coverage and reinfection in most populations, mild-disease autopsy sample from decedents with death long after infection and from unrelated causes are now nearly impossible to assemble prospectively.

In this post-mortem study conducted in Singapore, we examined lung, heart and brain tissues from four decedents, who recovered from mild COVID-19 more than 250 days earlier and later died of unrelated cause between February and May 2021. As these samples were obtained during the early phase of the national vaccination program, this cohort represents a unique opportunity to investigate tissue immunopathology of PACS with minimal confounding from vaccination before widespread vaccine uptake (5). We aimed to identify evidence of persistent inflammation in the post-mortem tissues and to explore molecular pathways contributing to PACS or asymptomatic long-term sequelae.

## Methods

### Case profiles

The post-mortem characteristics of the four cases are summarized in **Table 1**. Four male decedents aged 27–51 years with documented prior SARS-CoV-2 infection underwent mandatory post-mortem examination by the Forensic Medicine Division, Health Sciences Authority (HSA), Singapore, following unexpected or unnatural deaths between February and May 2021. Primary SARS-CoV-2 infection was documented by serology in cases 1–3 and by PCR in case 4, and the interval from initial positive test to death ranged from 258 to 355 days. All four cases experienced mild infection without the need for hospitalization, although details of acute symptoms were unavailable and the infecting viral strain was not documented. Post-mortem serology was also conducted despite unknown PACS symptom and status. Electronic health records showed no documented COVID-19 vaccination for two cases, while vaccination records could not be retrieved for the remaining two cases, precluding confirmation of their vaccination status.

**Table 1:** Cohort characteristics.

Category	Characteristic	Case 1	Case 2	Case 3	Case 4
Profile	Age/Sex	31/Male	28/Male	51/Male	27/Male
	Race	Indian	Indian	Chinese	Indian
	Pertinent medical history	None	None	Hypertension	None
COVID-19 History	Date of initial COVID-19 test	20/05/20	24/06/20	07/07/20	10/06/20
	Diagnostic COVID-19 test taken	Serology	Serology	Serology	PCR
	Hospitalization	No	No	No	No
	Acute symptom(s)	Not known	Not known	Not known	Not known
	PACS symptom(s)	Not known	Not known	Not known	Not known
Post-mortem	Date of death	01/02/21	22/04/21	04/05/21	30/05/21
	Cause of death	Industrial accident - thoracic and abdominal injuries	Road traffic accident - multiple injuries	Sudden collapse - stroke	Fall from height - multiple injuries
	Post-mortem COVID-19 test	Positive Serology	Positive Serology	Negative Serology	Specimen clotted, rejected
	Interval between initial to post-mortem COVID-19 test	258 days	303 days	302 days	355 days
	Tissues obtained	Lung Brain	Lung Heart	Lung Brain	Heart Brain

4-μm formalin-fixed, paraffin-embedded (FFPE) tissue sections were obtained from the Health Sciences Authority (HSA) for histopathological and molecular analyses. Sampling combinations included lung and brain tissues from cases 1 and 3, lung and heart tissues from case 2, and heart and brain tissues from case 4. Negative-control tissues were obtained from two individuals who died in 2022 and had no documented history of COVID-19.

### Immunohistochemistry

Immunohistochemistry (IHC) staining for SARS-CoV-2 nucleocapsid protein (Novus Biologicals, polyclonal) was performed on the FFPE tissue samples from all four cases, as previously described (2, 6). Appropriate positive and negative controls were included. Nuclei were counterstained with hematoxylin. Slides were scanned using a Zeiss Axioscan 7 (Zeiss, Oberkochen, Germany).

### Multiplex IHC

Multiplex IHC (mIHC) was performed on the tissue sections using a Leica Bond Max autostainer (Leica Biosystems, Melbourne, Australia). The Bond Refine Detection Kit (Leica Biosystems, Newcastle Upon Tyne, UK) and Panovue 7-plex TSA IHC Kit (Panovue, China) (7–11) were used. Sections were deparaffinized and rehydrated. They underwent repeated cycles of heat-induced epitope retrieval, incubation with primary antibodies, HRP-conjugated secondary antibodies (Bond Refine Kit), and tyramide signal amplification (Panovue, China). Primary antibodies used were SARS-CoV-2 nucleocapsid protein (Novus Biologicals, polyclonal), CD169 (Abcam, SP216), CD206 (Cell Signaling Technology, E6T5J), and CD68 (Agilent-Dako, PG-M1). DAPI (Panovue, China) was used for nuclear counterstaining.

Slides were mounted with ProLong Diamond Antifade Mountant (Molecular Probes, Life Technologies, USA) and cured at room temperature in the dark for 24 hours. Imaging was performed with a Zeiss Axioscan 7 and analyzed using HALO v3.6 (Indica Labs, Albuquerque, NM, USA).

### Single-sample gene set enrichment analysis

The ssGSEA2.0 repository was cloned (https://github.com/broadinstitute/ssGSEA2.0). MSigDB C8 gene sets (v7.2) were downloaded as gene symbols (http://www.gsea-msigdb.org/gsea/downloads.jsp) and filtered for single-cell-derived liver cell type signatures (12). The TPM gene expression matrix output by RSEM was converted to Gene Cluster Text (GCT) format. ssGSEA was performed using ssgsea-cli.R, generating enrichment scores for each sample. All gene sets passed an FDR-corrected p-value threshold of <0.05. Median scores and *Z*-scores were calculated for each cluster.

Cluster-specific gene sets were identified using one-vs-all Wilcoxon tests on ssGSEA enrichment scores, with FDR-adjusted p-values. Log fold change (logFC) was computed as the log_2_ ratio of the mean enrichment scores for the target cluster versus all other clusters.

### Digital spatial profiling

NanoString spatial profiling (13, 14) was used to simultaneously quantify two protein markers and 1,800 immune-related transcripts on FFPE tissue slides. Following deparaffinization and antigen retrieval, sections were incubated overnight with fluorescent-labeled antibodies against CD45 (Novus Biologicals, 2B11 + PD7/26), CD68 (Novus Biologicals, KP1), and PanCK (Novus Biologicals, AE1/AE3) to visualize morphological features and define regions-of-interest (ROIs). Slides were scanned using the GeoMx DSP instrument to generate digital fluorescent images for ROI selection.

High-resolution multiplex profiling was performed on ROIs classified as CD45^+^ (immune-cell-rich) or CD68^+^ (macrophage-rich). UV light was projected through a digital micromirror device (DMD) to selectively illuminate each ROI, cleaving photocleavable oligonucleotides (PC-oligos) from the probes. The released oligos were collected via microcapillary aspiration and transferred to a 96-well plate. Quantification was performed using the nCounter System (15, 16), and counts were normalized using the 75th percentile signal within each ROI (Q3 normalization) (17).

## Results

### Persistent SARS-CoV-2 antigen in lung and heart tissues

**Figure 1:**
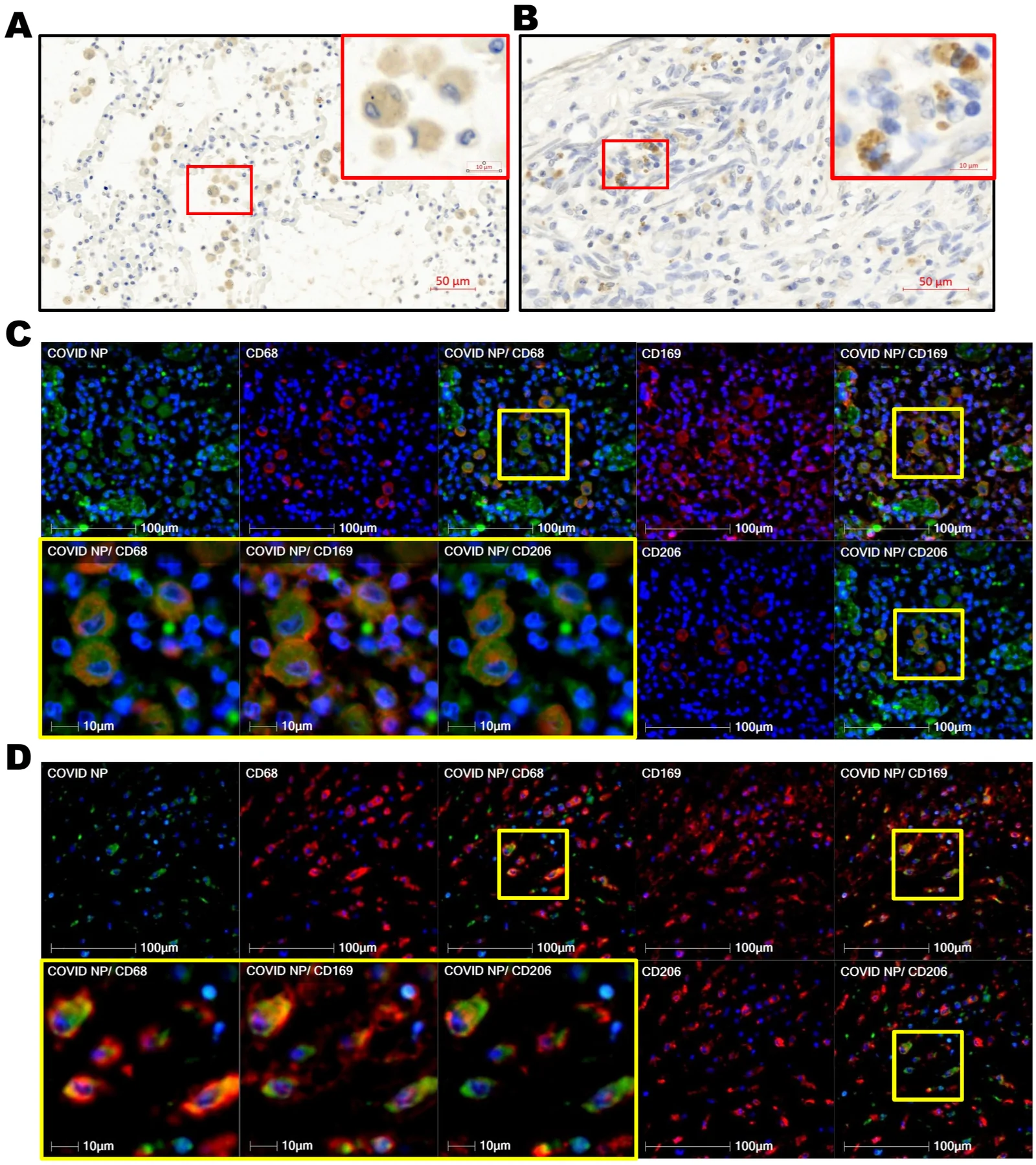
Persistence of SARS-CoV-2 nucleocapsid protein in infected lung and heart tissues. **(A, B)** Representative immunohistochemistry (IHC) for SARS-CoV-2 nucleocapsid protein (NP) infected **(A)** lung and **(B)** heart tissues. Brown cytoplasmic staining indicated NP, and blue hematoxylin counterstains the nuclei. Red boxes demarcate an area enlarged with scale bar at 10um to demonstrate a high-resolution field of view, visualizing positively stained cells for NP. **(C, D)** Representative multiplex IHC in infected **(C)** lung and **(D)** heart tissue for (blue) DAPI, (green) NP and (red) macrophage markers - i.e. CD68, CD169, CD206. Yellow square boxes demarcate an area enlarged with scale bar at 10um to demonstrate a high-resolution field of view, visualizing double positive co-localization between NP and a macrophage marker.

Post-mortem COVID-19 serology conducted 1 day after death was positive in cases 1 and 2, negative in case 3, and uninterpretable in case 4 due to sample clotting (**Table 1**). Using IHC, we detected SARS-CoV-2 nucleocapsid protein (NP) in lung and heart tissues from the four cases (**Figures 1A, B**, **Supplementary Figures 1A, B**). Knowing that NP co-localizes with macrophage markers (2, 6), we performed mIHC for NP and macrophage markers (CD68, CD169 and CD206) on the tissues. NP^+^ macrophages were detected in the lung tissue from cases 2 and 3 but not in case 1 (**Figure 1C**). In heart tissues, NP^+^ macrophages were detected in case 2 but not in case 4 (**Figure 1D**, **Supplementary Figures 1C, D**).

### Distinct tissue-specific immune modulation and differential gene expression

We next analyzed immune-rich regions of the lung, heart and brain using bulk RNA sequencing and GeoMx spatial transcriptomics to examine tissue-specific inflammation.

**Figure 2:**
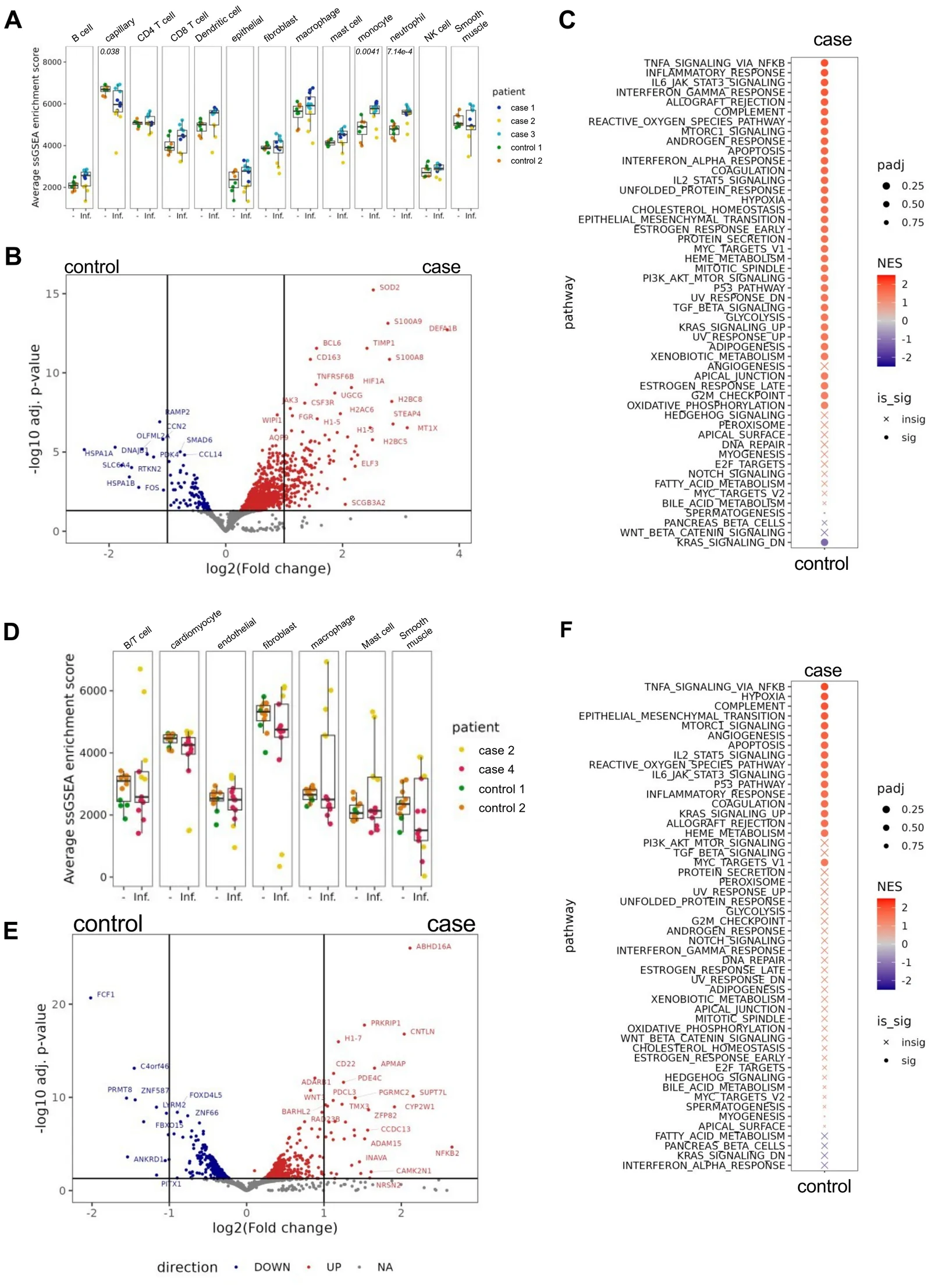
GeoMx digital spatial profiling of infected lung and heart tissues. **(A, D)** Single-sample gene-set enrichment analysis (ssGSEA) of Q3-normalized region-of-interest (ROI) transcript counts using MSigDB C8 cell-type gene sets; scores are in arbitrary units; 4–8 ROIs per **(A)** lung and **(D)** heart tissue. Each point represents one ROI and is colored by donor; boxes show the median and interquartile range, with whiskers extending to 1.5× the interquartile range. ‘−’ denotes control tissue and ‘Inf.’ denotes infected tissue. Values printed above selected facets are FDR-adjusted P values from Wilcoxon tests. **(B, E)** Volcano plot of differential gene expression in case versus control **(B)** of lung and **(E)** heart. The x-axis represents log2(fold change), and y-axis represents −log10(FDR-adjusted P value). Red and blue dots denote genes significantly upregulated in case and control tissue, respectively, while gray dots represent genes that did not meet both significance thresholds. Vertical and horizontal lines indicate |log2(fold change)| = 1 and FDR-adjusted *P* = 0.05. Selected differentially expressed genes are labeled. **(C, F)** Gene-set enrichment analysis (GSEA) of the ranked differential-expression results using MSigDB Hallmark gene sets in **(C)** lung and **(F)** heart tissues. Color encodes the normalized enrichment score (NES; positive/red, case enriched; negative/blue, control enriched); symbol size encodes the adjusted P value, with larger symbols indicating smaller values. Filled circles indicate FDR-adjusted *P* < 0.05 and crosses indicate non-significant gene sets. Each case is a biological replicate, and individual ROIs are nested spatial sub-samples.

In lung tissue (cases 1-3), ssGSEA of 12 CD45^+^ ROIs showed significant enrichment of monocytes and neutrophils (*P* < 0.005) (**Figure 2A**; **Supplementary Figure 2A**), suggesting an ongoing inflammatory response to persistent SARS-CoV-2, despite the lack of single-cell resolution. Differential gene expression analysis showed upregulation of *CD163*, *S100A8/9*, *TIMP1*, *DEFA1B*, *HIF1A*, *SOD2*, *BCL6* and *CSF3R* in infected lung tissue (**Figure 2B**). Hallmark GSEA identified NFκB–TNFα signaling, IL-6–JAK–STAT3 signaling, IFNγ response, complement activation and reactive-oxygen-species (ROS) pathways as significantly enriched in infected lung relative to controls (**Figure 2C**; **Supplementary Figure 2B**). Infected heart tissue (cases 2 and 4) similarly exhibited enrichment of immune cells (**Figure 2D**; **Supplementary Figure 2C**). Differential gene expression analysis identified upregulation of *ABHD16A* as the top hit, together with *APMAP*, *CD22*, *PDE4C*, *PDCL3*, *PRKRIP1* and *SUPT7L* (**Figure 2E**). Hallmark GSEA showed that immune-related pathways enriched in infected heart largely reflected those in lung, with additional enrichment of hypoxia and IL-2/STAT5 signaling among the top pathways (**Figure 2F**; **Supplementary Figure 2D**).

**Figure 3:**
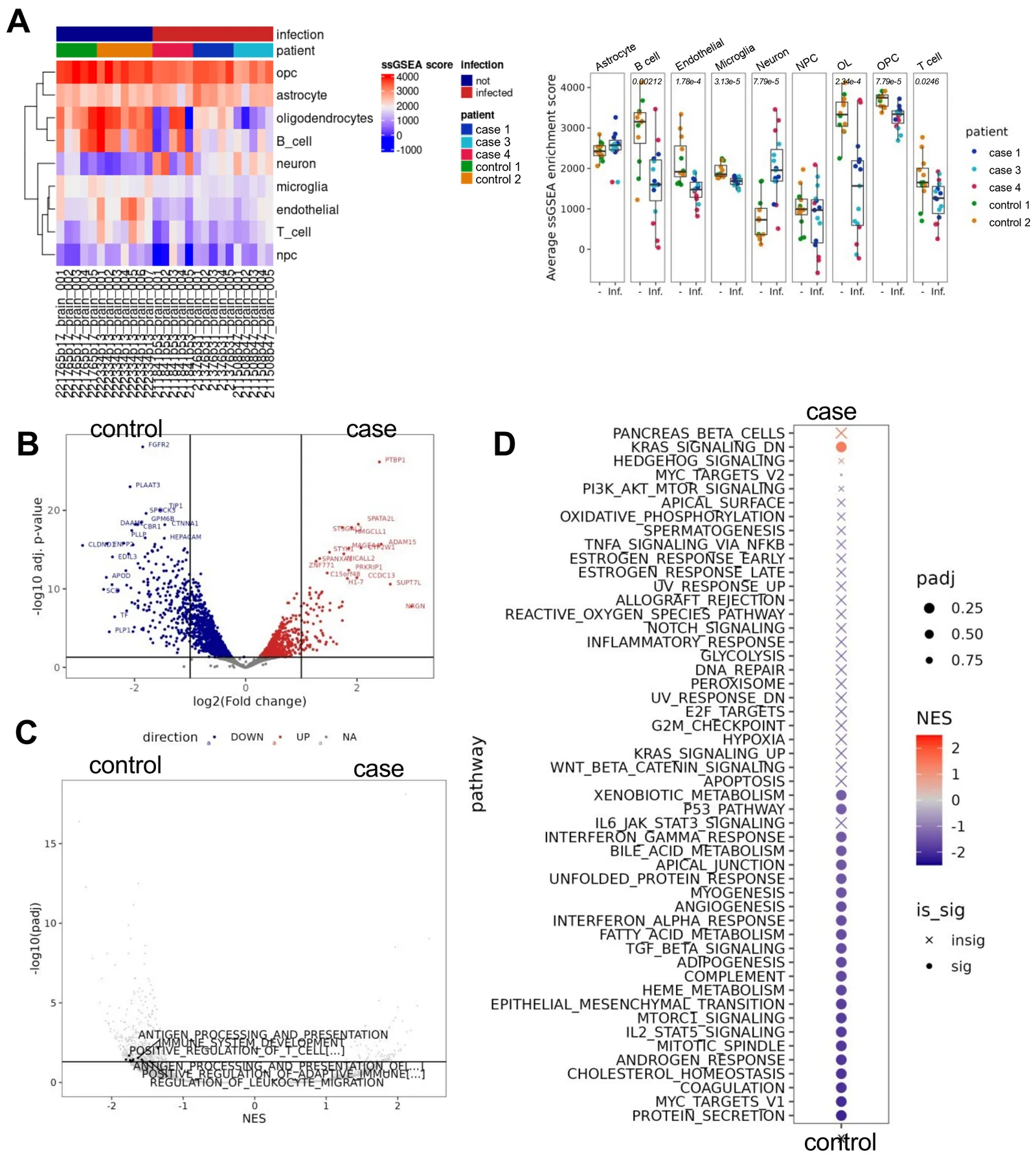
GeoMx digital spatial profiling of infected brain tissue. **(A)** Single-sample gene-set enrichment analysis (ssGSEA) of Q3-normalized region-of-interest (ROI) transcript counts using MSigDB C8 cell-type gene sets; scores are in arbitrary units; 4–8 ROIs per brain tissue. (right) Box and whisker plot; each point represents one ROI and is colored by donor; boxes show the median and interquartile range, with whiskers extending to 1.5× the interquartile range. ‘−’ denotes control tissue and ‘Inf.’ denotes infected tissue. Values printed above selected facets are FDR-adjusted P values from Wilcoxon tests. Oligodendrocyte precursor cell, OPC; neural progenitor cell, NPC. (left) Heatmap; rows depict cell-type signatures, columns are ROIs, and the top annotation bars indicates infection status and case ID. **(B)** Volcano plot of differential gene expression in case versus control brain. The x-axis represents log2(fold change), and y-axis represents −log10(FDR-adjusted P value). Red and blue dots denote genes significantly upregulated in case and control tissue, respectively, while gray dots represent genes that did not meet both significance thresholds. Vertical and horizontal lines indicate |log2(fold change)| = 1 and FDR-adjusted *P* = 0.05. Selected differentially expressed genes are labeled. **(C)** Gene-set enrichment analysis (GSEA) of the ranked differential-expression results using Gene Ontology Biological Process (GO: BP) gene set. The x-axis represents the normalized enrichment score (NES), and the y-axis represents −log10(FDR-adjusted P value); horizontal line indicates an FDR-adjusted *P* = 0.05. Positive NES indicates enrichment in case tissues while negative NES indicates enrichment in control tissues. Selected enriched gene sets are labeled. **(D)** GSEA using MSigDB Hallmark gene set. Color indicates NES (positive/red, case enriched; negative/blue, control enriched); symbol size shows the adjusted P value, with larger symbols indicating smaller values. Filled circles indicate FDR-adjusted *P* < 0.05 and crosses indicate non-significant gene sets. Each case is a biological replicate, and individual ROIs are nested spatial sub-samples.

In contrast to the lung and heart, infected brain tissues (cases 1, 3 and 4) showed limited signs of inflammation, with significantly reduced immune cells (**Figure 3A**). Although several immune-related genes (*PTBP1*, *SPATA2L*, *ST3GAL2*, *STYKI*) were upregulated (**Figure 3B**), the corresponding Hallmark immune-related gene sets were not enriched (**Figure 3C**). The only significantly enriched Hallmark gene set in the infected brain was associated with KRAS signaling (**Figure 3D**). Conversely, genes involved in structural strength, neuroprotection and immune regulation (*FGFR2*, *PLAAT3*, *TIP1*, *SPOCK3*) (**Figure 3B**), along with several inflammatory pathways (**Figures 3C, D**), were downregulated in infected brain tissue.

## Discussion

In this post-mortem analysis of lung, heart and brain tissues from four decedents, more than 250 days after mild COVID-19 and in the absence of documented PACS symptoms, we detected the presence of SARS-CoV-2 NP within macrophages in the lung and heart. This antigen persistence was accompanied by a coordinated inflammatory response, consistent with the self-sustaining inflammatory loop proposed for PACS (18). Additionally, brain tissue showed vascular dysfunction and altered neuroimmune homeostasis, suggesting that organ-specific tissue niches shape long-term inflammatory phenotype.

The detection of NP^+^ macrophages in lung and heart tissue is consistent with accumulating evidence that viral material may persist in macrophages (2–4, 6, 19). Mechanistically, the presence of viral antigen in macrophages may be due to distinct processes. Inflammatory monocyte-derived macrophages have been implicated in SARS-CoV-2-associated inflammatory injury, where antibody-opsonized virus can enter monocytes through Fc gamma receptors and trigger inflammasome activation and pyroptosis without requiring production infection (20). Macrophages, particularly those of ACE2^low^ expression, may acquire viral components through phagocytosis of infected cells or uptake of viral immune complexes (3), whereas ACE2^high^ alveolar macrophages demonstrated long-term viral persistence, supporting the possibility that resident macrophages may serve as reservoirs for viral persistence (21). While our proteomic finding aligns with previous reports, it only enabled the detection and localization of persistent viral NP but could not determine whether it remained replication competent or reflected productive viral replication. Nevertheless, persistent intracellular antigen may sustain inflammasome activation and pyroptosis, releasing viral RNA and proinflammatory cytokines that amplify inflammation through the recruitment of monocyte and macrophage – a hallmark of PACS (21, 22). Supporting this model, prior spatial transcriptomics studies have linked tissue-localized viral persistence to ongoing local immune activation (23).

Infected lung tissue exhibited differential gene expression, suggestive of a coordinated inflammatory response. Upregulation of *CD163* and *S100A8/9* could activate TLR4 and RAGE, promoting TNFα, CXCL8 and IL-6 secretion through NFκB signaling (24) — a pattern that has been formalized as a self-sustaining PACS inflammatory loop (18). *TIMP1* (25), *DEFA1B* (26), and *HIF1A* (27) are linked to systemic inflammation, neutrophil degranulation, and hypoxia, with HIF-1α further increasing IL-1β and IL-6 expression, reinforcing the proinflammatory feedback loop (18, 27). In response to inflammation, we also observed regulatory mechanisms via the upregulation of *SOD2* (28), *BCL6* (29) and *CSF3R* (30), which limits oxidative stress, macrophage activity and IL-6 production. GSEA findings of enriched NFκB–TNFα, IL-6–JAK–STAT3 signaling, IFNγ response, complement activation and ROS pathways correlated with the gene expression observation. Particularly, persistent complement activation has been reported at least 6–12 months post-COVID and in individuals with PACS (31), highlighting its potential as a therapeutic target. Woodruff et al. further demonstrated that inflammatory PACS can persist beyond 1 year after infection, characterized by high levels of neutrophil activity and IL-1β/IL-6/IL-8-associated inflammatory signals (32). Together, these blood-based findings provide an independent framework supporting the self-amplifying inflammatory circuit suggested by our lung tissue data, in which neutrophil activity, complement dysregulation, myeloid inflammation, and tissue injury may collectively sustain immune activation after resolution of acute infection. Molecules targeting S100A8/9–TLR4/RAGE signaling and T-cell exhaustion pathways also represent rational target candidates, consistent with frameworks outlined in recent long-COVID mechanism review (33, 34).

Among the genes altered in infected heart, *ABHD16A* emerged as the top differentially expressed gene. As a regulator of lipid metabolism and immune signaling via ABHD12, it modulates macrophage cytokine release and may contribute to sustained cardiac inflammation (35, 36). In addition, upregulation of *APMAP* (37), *CD22* (38), *PDE4C* (39), and *PDCL3* (40) which are associated with macrophage phagocytic activity and an M2 phenotype, alongside *PRKRIP1*, a negative regulator of PKR, involved in inflammasome activation and pyroptosis (41), suggests a compensatory response to chronic inflammation. Infected heart tissue revealed additional enrichment for hypoxia and IL-2/STAT5 signaling. Hypoxic tissue microenvironments are known to sustain hyperinflammation in PACS through NFκB activation and HIF-1α signaling (18, 27, 42), and PACS-associated cardiometabolic dysfunction has been linked to elevated CCL5, a mediator of HIF-1α (42). Systemic hypoxia may itself result from carotid body dysregulation due to immune-mediated destruction of glomus cells and persistent local inflammation (43). The IL-2/STAT5 enrichment is also consistent with PACS, as immune profiling described elevated intracellular IL-2 in T cells (44). While IL-2 promotes T-cell expansion, chronic IL-2 exposure may also induce T-cell exhaustion, as demonstrated by the reduction of CD4^+^ central memory T cells and increased exhausted CD4^+^ T cells (44). Large-scale post-hospitalization plasma phenotyping, however, revealed an inverse correlation between IL-2 levels and cardiorespiratory symptoms (45). Given that several heart-tissue DSP signals were predominantly driven by individual patients rather than from cohort average, the heart-specific findings should be regarded as exploratory and require validation in larger, independent cohorts (46).

In contrast to the lung and heart, infected brain tissues exhibited reduced immune cells with significant enrichment of the KRAS signaling pathway. KRAS activation has been implicated in increased vascular permeability and endothelial barrier dysfunction following SARS-CoV-2 infection (47), and may contribute to impaired adaptive immunity and coagulation abnormalities in PACS-related cognitive dysfunction (48). The downregulation of *FGFR2*, *PLAAT3*, *TIP1* and *SPOCK3* (49–52), together with suppression of several inflammatory pathways, suggests an attenuated response that may reflect viral modulation. Importantly, reduced immune cell abundance does not preclude neurological PACS-associated pathology, as neurological manifestations were associated with blood-brain barrier disruption, endothelial modulation, coagulation dysregulation and altered systemic immunity rather than prominent local immune cell infiltration (48). Thus, the brain-specific molecular profile observed in our cohort may plausibly represent a distinct tissue response characterized by vascular dysfunction and altered neuroimmune homeostasis. These findings should be read against the still-limited understanding of COVID-19-associated neurological complications.

Overall, the distinct tissue-level profiles observed across tissues suggest that persistent viral antigen elicits organ-specific immune and molecular response, underpinning distinct manifestations that may contribute to PACS. A particular strength of this study lies in its unique and now irreplaceable cohort, rather than in analytical novelty. Tissue samples were collected during the early phase of the national vaccine program, before widespread vaccine uptake and recurrent infection. This provides a unique reference for defining the unmodified disease pathogenesis. Such a cohort can no longer be assembled prospectively and thus, our cohort represent a biological baseline that enables the investigation of persistent viral antigen and tissue inflammation following mild COVID-19, independent of severe acute disease, reinfection, or documented vaccination. Additionally, the male-only composite reduced variability related to sex-specific immune differences. Notably, in contrast to early epidemiological studies which established PACS as a multisystem disorder (53), our findings extend these observations by providing tissue-level evidence that persistent viral antigen and spatially organized immune activation can remain detectable in specific organs, more than 250 days after mild acute disease. It establishes a valuable pathological reference that can be interpreted for vaccinated and reinfection-rich cohorts.

Nevertheless, several limitations should be acknowledged. Despite the uniqueness of our cohort, the small sample size limits statistical power and generalizability across broader populations. Additionally, due to the circumstances of death, detailed antemortem clinical information, including PACS status and symptom assessment was unavailable hence, we cannot directly link specific tissue-level pathological findings to individual symptom and disease trajectory. Furthermore, although samples were collected during early phase of the vaccine rollout, vaccination status could not be confirmed for all individuals, introducing a potential source of residual confounding. On technicality, DSP analyses were limited to 4–8 ROIs per tissue, with each case treated as the biological replicates and ROIs as nested spatial sub-samples; the molecular findings should be considered as exploratory and hypothesis-generating observations rather than population-level estimates. Variable forensic tissue availability limited uniform sampling which restricted within-person cross-organ comparisons. Lastly, since the study relied on proteomic techniques to detect viral antigen and determine localization, it was unable to assess the replication competency of the persistent virus. Further studies involving functional assays and subgenomic RNA detection would be necessary to determine the presence of productive viral replication.

Overall, the concordance of our findings with independent tissue-level studies, together with the uniqueness of our post-mortem cohort, supports the persistence of SARS-CoV-2 antigen and organ-specific immune dysregulation more than 250 days after mild infection, even in the absence of documented PACS symptoms. By profiling lung, heart and brain, we reveal distinct patterns of sub-clinical post-acute tissue response that provide a rare reference for understanding the unmodified consequences of SARS-CoV-2 infection that may contribute to PACS.

## Data Availability

The raw data supporting the conclusions of this article will be made available by the authors, without undue reservation.

## Supplementary material

Supplementary Figure 1 Controls for SARS-CoV-2 nucleocapsid protein detection and macrophage co-localization. **(A, B)** Representative immunohistochemistry image with NP-negative **(A)** lung and **(B)** heart tissues. The lack of brown cytoplasmic staining indicates an absence of NP in the tissue, while blue hematoxylin counterstains the nuclei of cells. **(C, D)** Representative multiplex IHC images for **(C)** lung and **(D)** heart tissue for (blue) DAPI, (green) NP and (red) macrophage markers - i.e. CD68, CD169, CD206. The lack of green staining in **(C, D)** indicates the absence of NP in the tissue. Yellow square boxes demarcate an area enlarged with scale bar at 10um to demonstrate a high-resolution field of view, visualising single positive macrophage marker staining with no demonstrative of co-localised signals.

Supplementary Figure 2 Cell-type and Gene Ontology pathway enrichment in post-mortem lung and heart tissue. **(A, C)** ssGSEA of Q3-normalized ROI transcript counts using MSigDB C8 cell-type gene sets in lung **(A)** and heart **(C)**; scores are in arbitrary units. Rows are cell-type signatures, columns are ROIs, and the top annotation bars identify infection group and donor. **(B, D)** GSEA of ranked differential-expression results using Gene Ontology Biological Process (GO:BP) gene sets in lung **(B)** and heart **(D)**. The x-axis is the normalized enrichment score (NES), the y-axis is −log10(FDR-adjusted P value), and the horizontal line marks FDR-adjusted P = 0.05; positive NES indicates case enrichment, and negative NES indicates control enrichment. Black points pass the FDR threshold, gray points do not and selected significant gene sets are labeled. The lung analysis comprised three case donors and two control donors; the heart analysis comprised two case donors and two control donors. Each tissue sample contributed 4–8 ROIs. Each case is a biological replicate, and each CD45-positive immune-cell-rich or CD68-positive macrophage-rich ROI is a nested spatial sub-sample.
